# Mimicking Alveolar Lung Structures with Lattice Designs

**DOI:** 10.3390/polym17192572

**Published:** 2025-09-23

**Authors:** Aniello Riccio, Angela Russo, Andrea Sellitto, Maria Rosaria Barillari, Alfonso Reginelli, Salvatore Cappabianca

**Affiliations:** 1 Department of Engineering, Luigi Vanvitelli University, Via Roma 29, 81031 Aversa, Italy; 2 Audiology and Phoniatrics Unit, Department of Mental and Physical Health, Luigi Vanvitelli University, 80138 Naples, Italy; 3 Department of Precision Medicine, University of Campania, 80138 Napoli, Italy; 4 Department of Medical-Surgical and Odontostomatological Specialties, University of Campania, 80138 Naples, Italy

**Keywords:** lattice structure, additive manufacturing, finite elements method, stereolithography, surgical planning

## Abstract

Advances in additive manufacturing (AM) have revolutionized various sectors, including aerospace engineering, where the use of lattice structures has enabled the development of lightweight high-performance components with optimized mechanical properties. Building on these engineering principles, this study explores the application of aerospace-derived lattice design strategies to the biomedical field, specifically for the replication of human lung alveolar structures. The objective is to create anatomically accurate 3D-printed lung models suitable for surgical planning. Finite element analyses have been conducted using a CAD model of adult lungs, including the application of lattice structures generated through nTopology software, to evaluate the elasticity and density, critical for simulating lung mechanics. A preliminary prototype has been produced using stereolithography and flexible resin, showing the potential for realistic tactile feedback.

## 1. Introduction

In recent decades, the manufacturing industry has experienced a major revolution with the advent of additive manufacturing (AM) technology [1,2,3]. This production approach, commonly known as 3D printing, represents a significant breakthrough in the industrial scene and offers infinite opportunities in various sectors. Among the numerous applications of AM, one of the most promising areas of research and development is in the medical field. Indeed, this methodology, in contrast to traditional subtractive and formative processes, provides unique flexibility in component design and production, ensuring high precision, customization, and versatility [4,5]. These characteristics play a crucial role in the medical field, where the need for tailored solutions and high precision is a constant. Within the field of medicine, AM has led to numerous revolutionary developments. Indeed, its use ranges from the design of personalized prosthetics to the creation of anatomical models for educational and advanced surgical planning purposes. An extensive literature background can be found on this topic. The work in [6] deals with the use of 3D printing to improve preoperative planning in the treatment of resectable pancreatic adenocarcinoma. Study [7] demonstrates that 3D printing improves surgical planning for complex double-outlet right ventricle by better predicting the feasibility of biventricular repair, selecting optimal surgical techniques, and aiding preoperative discussions with families and specialists. Assia-Zamora et al. in [8] highlight the use of 3D printing (3DP) to plan and execute complex hepatobiliary surgeries, specifically for congenital portosystemic shunts (CPSs) in children. In [9], a study on a cost-effective workflow for three-dimensional modeling and printing (3DMP) of complex congenital heart defects is presented. Study [10] evaluates the use of a customized 3D-printed surgical guide to improve the accuracy and efficiency of external ventricular drain (EVD) placement by junior neurosurgeons. The guide facilitated preoperative trajectory planning and insertion, resulting in a 100% first-pass success rate and 93% optimal placement accuracy in 14 patients, including those with distorted ventricular anatomy. In [11], a mini-review is presented that explores the transformative impact of 3D printing on surgery, emphasizing its evolution from anatomical modeling to creating custom implants, prosthetics, and bio-printed tissues.

To fully exploit the potential of AM in the medical context, it is essential to understand the most common additive technologies for surgical planning and related materials for this use. The synergy between these two elements, such as technology and materials, indicates a new era in the future of medicine, with the promise of improving the quality of healthcare on a global scale. In this context, combining aerospace engineering findings in additive manufacturing technologies and innovative materials [12] with 3D printing for surgical planning can lead to significant advances in this field. This interdisciplinary collaboration can enable advances in areas such as complex reconstructive surgery, minimally invasive procedures, and regenerative medicine. For example, the use of aerospace-derived materials that have superior durability and mechanical properties can lead to the production of implants with greater longevity and functionality. Similarly, advanced aerospace AM technologies, such as multi-material printing and high-resolution fabrication, can improve the accuracy and utility of surgical planning models, helping surgeons to design optimal approaches for complex cases. Moreover, this multi-disciplinary approach may potentially reduce manufacturing costs and simplify manufacturing processes, making medical innovations more accessible, even in resource-limited contexts. The use of aerospace technologies and materials has very ancient roots. Among these technologies, lattice structures represent a powerful design tool now being adapted to mimic the complex micro-architecture of soft human tissues.

The text in [13] discusses the utilization of NASA technology and its application to medicine, highlighting how new or improved commercially available medical products can incorporate aerospace technology. A bipolar donor–recipient model for medical technology transfer is presented to outline the methodology, which aims to identify medical problems and NASA technologies that together offer opportunities for successful medical products, secure early industry involvement in the transfer process, and gain acceptance from the medical community. Study [14] explores how aerospace materials, particularly composites, could have direct applications in orthopedic practice due to their advanced material properties. The article in [15] discusses the role of biomedical application teams funded by NASA that aim to apply aerospace technology to address critical problems in biomedical research and clinical medicine.

In the context of highly detailed 3D-printed anatomical models for surgical planning, the role of materials is crucial to ensure that the models are not only accurate in terms of size and geometry but also offer realistic tactile feedback for the simulation of surgical operations [16,17]. The printed models must reproduce the anatomical structure of the organ with high precision. The materials used must be able to imitate the density, texture, and surface of real biological tissues, such as skin, muscle, bone, and soft tissue. Surgeons must be able to feel and manipulate the model as if it were a real organ. Hence, the used materials need a tactile response that simulates the behavior of human tissue. For example, organ models must be rigid enough to represent structures such as bones, but also soft enough to simulate more elastic tissues such as muscle or visceral tissues.

Despite significant advances in 3D printing, there are still substantial limitations, especially when it comes to the flexibility of materials. For example, the lungs are organs that continuously expand and contract during respiration, so they have a “superelasticity” that allows them to adapt to the change in volume and pressure without losing their shape or integrity. Their ability to expand and adapt to the dynamics of respiration is one of the most difficult features to replicate in 3D-printed models. The lungs have a relatively low density compared with other organs in the human body due to their air-rich structure. Each lung contains millions of air-filled alveoli, which make up most of the lung volume. Replicating the complexity of the lungs internal structures requires advanced 3D printing techniques. This work deals with the mimicking of the alveolar structure of the lungs using lattice structures. A numerical model of the lungs has been developed in the finite element software Abaqus CAE and used to perform simulations comparing the behavior of human lung tissue with that of a commercially available flexible resin. This study aimed to identify an optimal solution, guided by design for additive manufacturing (DfAM) principles, to produce 3D-printed lungs that closely replicate the density and elasticity of real organs [18,19,20].

In Section 2 the finite element model of the lungs is presented, including the boundary conditions. In Section 3, the numerical application of lattice structure to the model is reported. Section 4 shows the preliminary production process, including thorough additive manufacturing. Finally, Section 5 reports a sensitivity analysis obtained by varying the lattice cells.

## 2. Clinical Specialties Using 3D Printing

Three-dimensional printing has been increasingly adopted across numerous clinical specialties for device manufacturing, anatomical modeling, and procedural support, with the most widespread applications centered on patient-specific implants and surgical instruments. Systematic reviews report extensive use in orthopedics, cardiovascular medicine, and maxillofacial and dental surgery, as well as in other surgical fields where complex anatomy and the need for individualized solutions provide substantial clinical benefits [21]. In cardiology, patient-specific heart and vascular models are employed to plan complex congenital and structural interventions and to simulate procedures such as valve repair or aortic surgery [22]. In orthopedics, applications include anatomical models, patient-specific cutting and drilling guides, and custom implants for reconstructive and limb-salvage procedures [23,24]. Maxillofacial and ENT surgery widely benefit from virtual surgical planning [25], patient-specific implants, and prebent fixation plates for mandibular, maxillary, and orbital reconstruction. In dentistry and endodontics, guided endodontic and implant templates enhance treatment precision in challenging anatomical scenarios. More broadly, neurosurgery, craniovertebral, ENT, and oncologic reconstructive procedures have demonstrated the value of 3D-printed models [26,27,28] and surgical guides [29,30] in improving resection accuracy and facilitating reconstruction. To better contextualize the clinical translation of additive manufacturing, Table 1 summarizes representative device categories, examples of their application, associated clinical benefits, and supporting evidence from the literature.

Building on this framework, the lattice-based lung models proposed in the present study can be envisioned as future tools for clinical workflows, particularly in surgical planning and patient-specific simulation, where the ability to reproduce realistic tactile feedback and anatomical fidelity could significantly enhance preoperative training and decision making. At the same time, their translation into practice will require further work on material optimization, biomechanical validation, and workflow integration to ensure that such models not only demonstrate technical feasibility but also deliver tangible value in real-world healthcare settings.

## 3. Numerical Model

**Figure 1:**
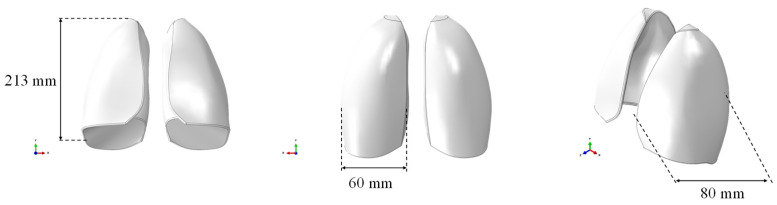
CAE model of the lungs.

The respiratory system was selected as a case study due to its complexity in terms of density and elasticity mimicking. Specifically, a solid CAD model of the lungs, without any internal alveolar structure, based on the size of the lungs of an adult male, was created and used for numerical simulations. The model was imported in the finite element software ABAQUS CAE, as shown in Figure 1, where the geometric dimensions are also reported. The CAE model consisted of both lungs represented as a single, solid, and internally filled part. It was prepared for discretization by making the required cuts to accommodate the mesh.

**Figure 2:**
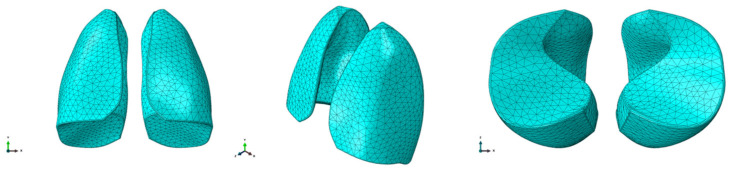
FEM model of the lungs.

Once the geometry was prepared, meshing was performed using C3D10M (quadratic formulation) elements. The ten-node tetrahedral element C3D10 is a general purpose tetrahedral element with four integration points. Elements of approximatively 10 mm size were considered to discretize the model. The final mesh was composed of 36,721 elements and 55,254 nodes. The finite element model is shown in Figure 2.

A solid homogeneous section was created to assign the proper material properties to the lung model. The elastic modulus of the lungs was taken from the literature [16], and for the density value [17]. Then, the section was assigned to the CAE model through the “Section Assignment” command. The elastic material properties of the lungs are reported in Table 2, including Young’s modulus E, Poisson’s modulus ν, and density ρ. The material hardness was measured using the Shore A scale, which is commonly used to characterize the hardness of flexible elastomeric materials, rubbers, and soft plastics. Shore A values range from 0 (very soft) to 100 (very hard), providing an indication of the material’s resistance to indentation and its flexibility. In this study, the Flexible 80A resin was used, representing a relatively soft material suitable for replicating the tactile properties of biological tissues.

Subsequently, the model was transferred to the “Assembly” module to assign the proper boundary conditions for the analyses. The purpose of the numerical simulations was to evaluate the expansion, in terms of displacements, of the lungs during the air inhalation phase. A dynamic explicit analysis type was preferred, which allowed for the inclusion of the time variable.

**Figure 3:**
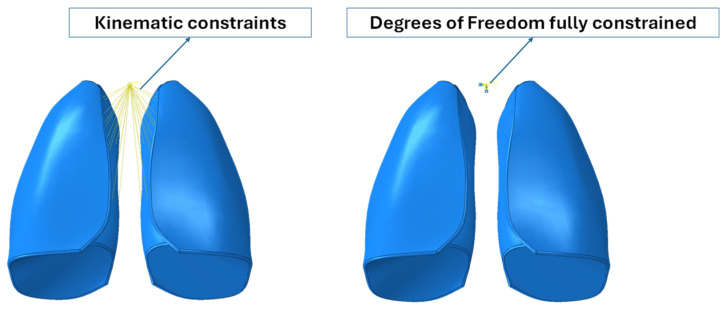
Boundary conditions.

Because the numerical model did not include the trachea and bronchi, kinematic constraints were implemented that connected the inner lateral surfaces of the lungs to a control point, which represented the larynx. Moreover, the degree of freedom of the reference points were fully constrained. Boundary conditions are shown in Figure 3.

**Figure 4:**
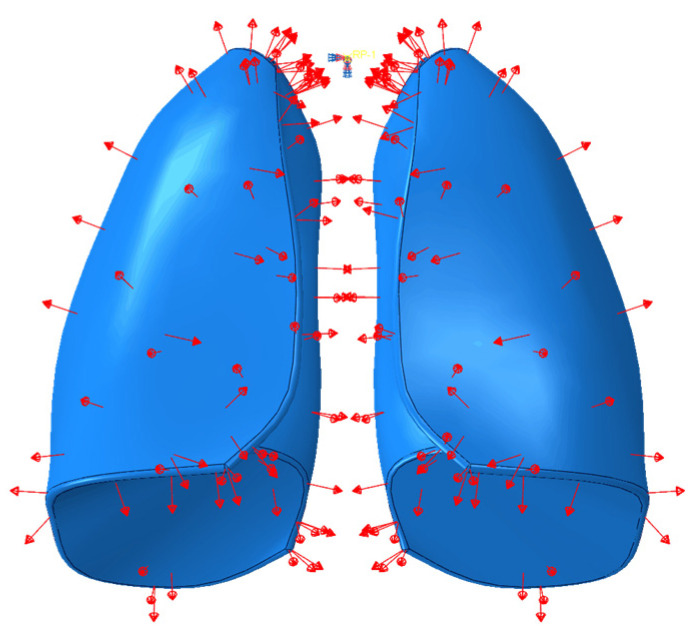
Pressure applied on the lungs’ surface.

With the aim to simulate breathing, particularly the phase of air inhalation inside the lungs, an explicit analysis was carried out in which a uniformly distributed depression was applied for a time of 1.3 s, which was equal to the time required for the lungs to introduce air. A pressure of 5900 Pa [20], that was uniformly distributed, was applied to the model. However, an amplitude was defined, so that the pressure was applied gradually over the 1.3 s of analysis time. In Figure 4 the pressure load applied on the lungs model is reported.

### Results

**Figure 5:**
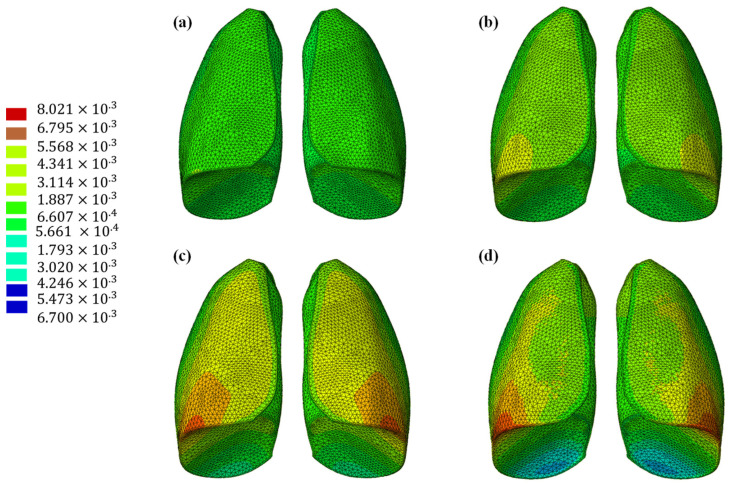
Out-of-plane displacement of the lung at different time instants: (**a**) 0.325 s, (**b**) 0.65 s, (**c**) 0.975, and (**d**) 1.3 s.

**Figure 6:**
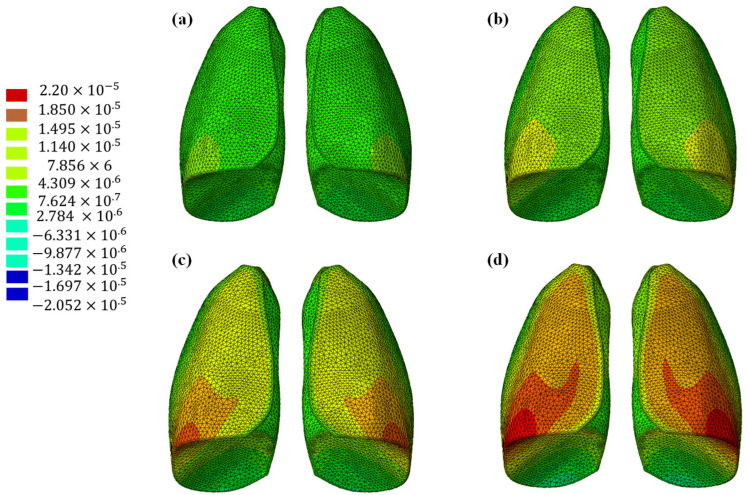
Out-of-plane displacement of the lung (Flexible 80A resin) at different time instants: (**a**) 0.325 s, (**b**) 0.65 s, (**c**) 0.975, and (**d**) 1.3 s.

The results of the numerical analysis conducted on the lung model are shown in Figure 5 and Figure 6, which display contour plots of the deformation in terms of out-of-plane displacement at different time instants (0.325, 0.65, 0.975, and 1.3 s). In Figure 1, the model is analyzed using the specific elastic properties of the lung tissue, listed in Table 2, while in Figure 2, the elastic properties of the Flexible 80A resin [21] are assigned to the model. The resin properties are reported in Table 2. The comparison clearly shows how the orders of magnitude of the displacement values are not comparable; the resin, despite being one of the most flexible available on the market, cannot faithfully reproduce the elastic behavior of the lung.

**Figure 7:**
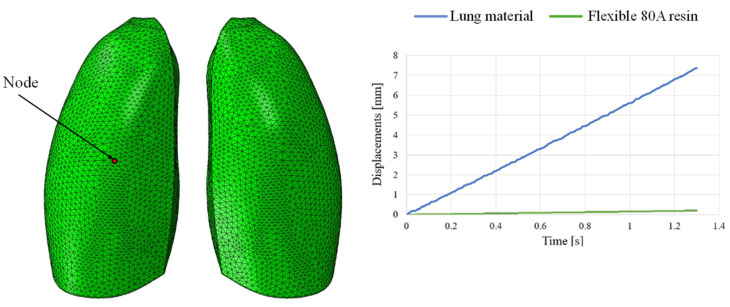
Displacement comparisons in one node of the model.

This discrepancy is confirmed by observing Figure 7, which shows the out-of-plane displacement of a representative node located at the center of the lung surface, as indicated by the red dot in the figure, for both configurations. In the graph, the out-of-plane displacements along the Y direction are plotted on the vertical axis, while the characteristic respiratory time is represented on the horizontal axis, allowing a direct comparison of the dynamic response between the two configurations. The graph clearly shows that the model assigned with the elastic properties of real lung tissue exhibits significantly larger displacements compared with the one made of Flexible 80A resin, confirming the inability of the resin to replicate the highly compliant behavior of pulmonary tissue. This result highlights a fundamental limitation: when considering both models as solid (i.e., fully filled), there is currently no 3D printable material capable of reproducing the highly elastic behavior of lung tissue. For this reason, a different strategy is required.

A solution comes from engineering, and specifically from design methodologies developed in the context of additive manufacturing, which exploit the concept of filling volumes with recursive internal structures known as lattice cells. These architectures enable the tuning of global mechanical properties by controlling the geometry and topology of the internal infill, offering a promising route to approximate the mechanical behavior of soft and complex biological tissues, such as the lung.

## 4. Lattice Structure Infill

**Figure 8:**
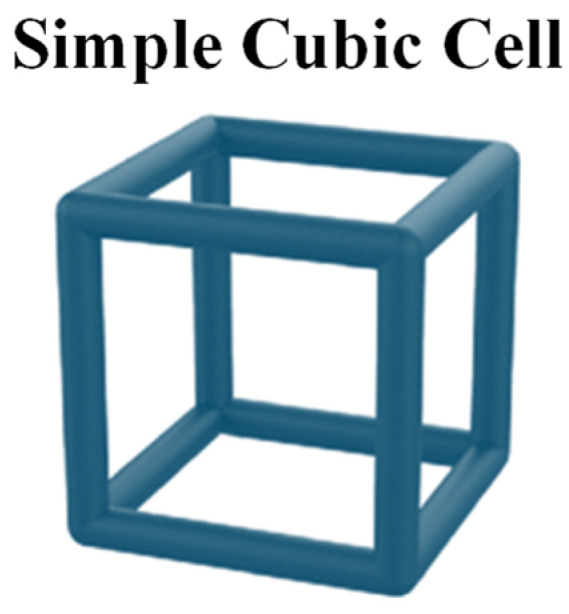
Simple cubic (SC) lattice cell.

This section presents the optimization of lungs performed according to the rules of DfAM. Indeed, lattice cells, which are typical AM structures strongly used in the aerospace and transport industries in general, have been used to reproduce the internal alveolar structure of the lung. To verify whether this approach results in a behavior closer to the real one in terms of elasticity, nTopology (nTop) software was used for this purpose. Indeed, nTop is an advanced generative design and engineering software that focuses on the creation of complex customized geometries for engineering applications. Lattice structures in various arrangements with different unit cell topologies can be generated using nTop. Among others, simple cubic (SC) lattice cells, as shown in Figure 8, have several advantages, primarily due to their geometric simplicity and symmetry properties, making them useful in various applications.

**Figure 9:**
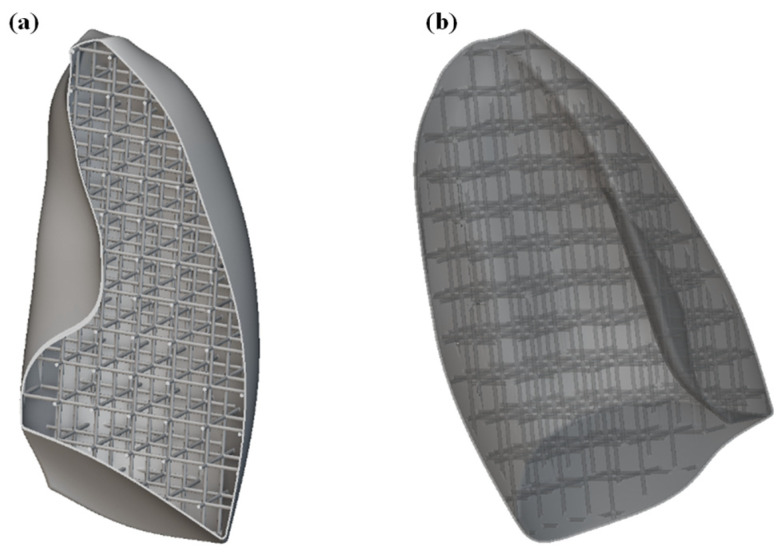
(**a**) Section view of the lung model showing the internal lattice structure; (**b**) complete lung model including the external shell and internal lattice infill.

The first step was to import the CAD model of the lungs within the nTOP software. For simplicity and to reduce the computational cost, only one of the lungs was considered. Subsequently, the solid lung model was voided by generating a 1 mm thick shell, compatibly with 3D printing capabilities. The interior of the lung was then filled with SC cells of size 20 × 20 × 20 mm, with a thickness of 2 mm. The image of the internal part of the lung is shown in Figure 9a, while the entire lung, including shell and lattice infill, is shown in Figure 9b.

**Figure 10:**
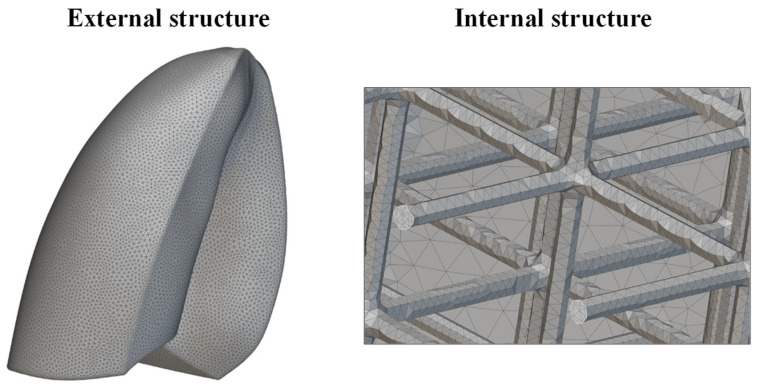
Mesh of the lung.

The new lung model was meshed using tetrahedral elements. An edge length of 2 mm was considered. A very fine mesh was needed to properly discretize the internal lattice structure, as shown in Figure 10. This level of detail was essential to correctly capture the mechanical response of the lattice during the simulation. The increased mesh density, while computationally demanding, allowed for a more realistic approximation of the deformation behavior of the infilled lung model. Finally, the mesh model was exported in compatible format and imported into Abaqus CAE software for finite element analysis, preserving the integrity of both the outer shell and internal lattice geometry throughout the simulation workflow.

Numerical analysis was performed, considering the same boundary conditions of Figure 3, including the fixed point, representative of the larynx, and loading conditions of Figure 4, which considered a uniformly distributed surface depression, mimicking breathing. Material properties of the Flexible 80A resin, in Table 2, were considered. In contrast to previous analyses, only one lung was taken into account to reduce the computational effort due to the very fine mesh size resulting from the presence of the lattice structure.

**Figure 11:**
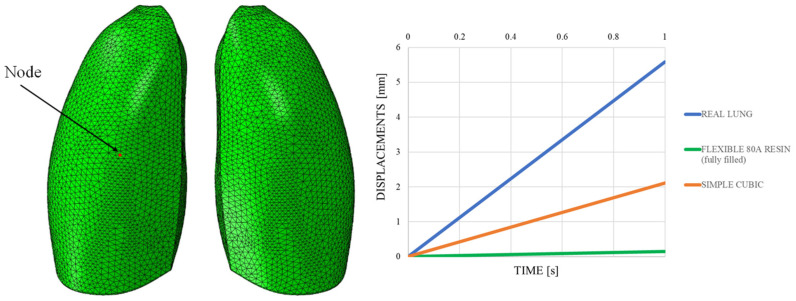
Displacements vs. time; simple cubic cell.

The out-of-plane displacement trend comparison shown in Figure 11, measured at a representative node positioned at the center of the lung model (red dot in Figure 11), reported as a function of the typical respiratory time, clearly demonstrates that the introduction of the lattice structure significantly improved the elastic response of the system, making it more comparable to that of real lung tissue. Nevertheless, it remains evident that the level of elasticity achieved is still considerably lower than the superelastic behavior that characterizes actual pulmonary tissue.

Although the combination of the selected resin and the adopted lattice infill improved the mechanical response of the model, it was still insufficient to faithfully replicate the complex behavior of real lung tissue. Both the material properties of the resin and the specific lattice configuration currently represent limiting factors. Nevertheless, the proposed approach remains promising and lays the groundwork for future developments.

## 5. Prototype 3D Printing

To further validate the proposed design approach and assess its feasibility from a manufacturing point of view, a preliminary 3D printing phase was carried out. This phase aimed to evaluate the printability, structural integrity, and tactile properties of the lattice-filled lung model using the selected resin and geometry.

**Figure 12:**
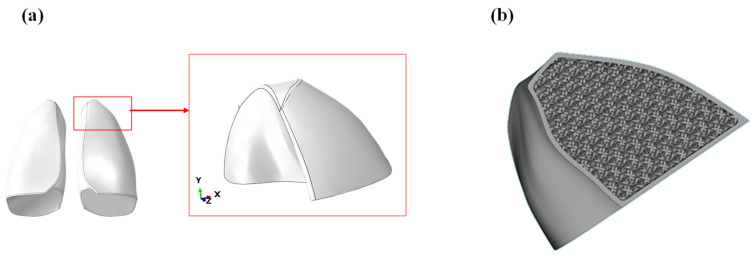
(**a**) Model of the lung lobe; (**b**) lobe filled with lattice structure.

A first preliminary print was performed considering only the upper lobe of the lung, as shown in Figure 12a. The printing was carried out using a Formlabs 3BL stereolithography printer, specifically designed to produce medical objects as it supports biocompatible materials. The CAD model of the lobe was imported into the software Materialize Magic, which allowed to create a lattice structure from a solid. Using this function, a characteristic outer thickness of the lung was defined, while the internal was filled with a lattice structure using the lattice structure shown in Figure 12b. Each cell was 20 × 20 × 20 mm.

**Figure 13:**
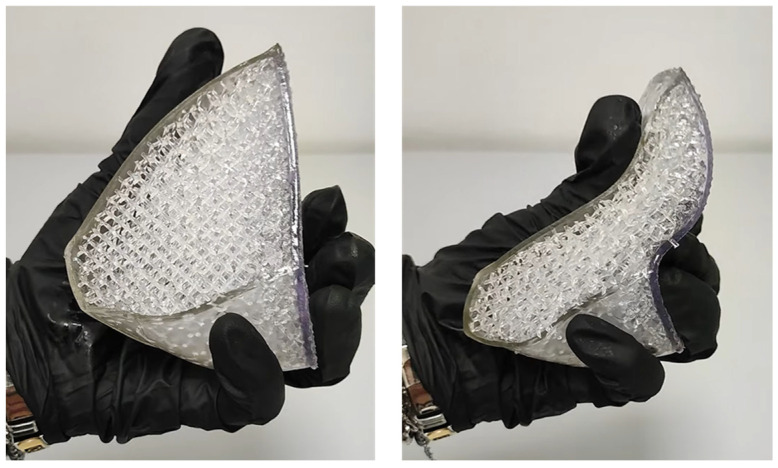
Preliminary printing of a lung lobe.

The lung lobe model was transferred to Form 3BL software for printing, named PreForm, to choose the model orientation, define the supporting structures, and perform the slicing. Flexible80A resin was used for the printing, which was characterized by a hardness Shore value of 80 and supported a layer thickness of 0.1 mm. The image of the printed part, highlighting its flexibility, is shown in Figure 13.

The polymerization (curing) of the Flexible80A resin used in this study was performed according to the manufacturer’s recommendations, specifically 10 min at 60 °C.

**Figure 14:**
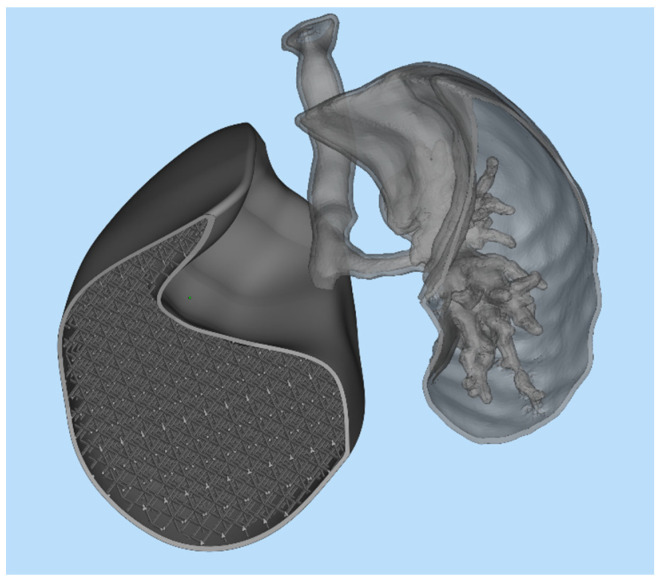
Demonstrator structure.

After completing the test print of the lung lobe, a demonstrator model was developed to visually and functionally highlight the potential of the proposed approach. This model combined two distinct representations of the lung: on one side, the anatomical geometry reconstructed from DICOM images of an adult male patient; on the other side, the CAD-generated lung model filled with a simple cubic lattice infill, as previously shown in Figure 10. The two geometries were merged into a single STL mesh, creating a hybrid structure that allowed a direct visual comparison between the real anatomical morphology and the engineered lattice-based design. As illustrated in Figure 14 the model was cut along the lower section to expose the internal components on the left side and the bronchial tree on the right. This composite model was then imported into PreForm, the slicing software for the Form 3BL printer, to define the build orientation and generate the required support structures for fabrication.

**Figure 15:**
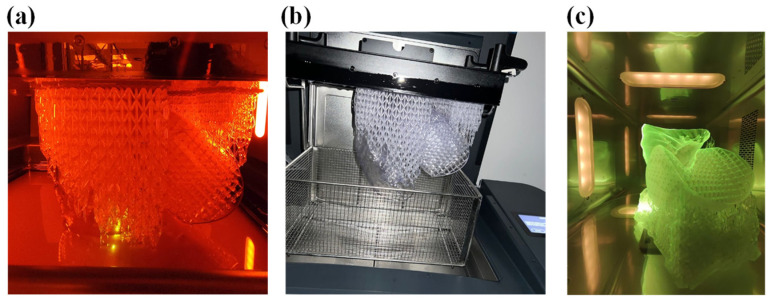
(**a**) Printing of the demonstrator, (**b**) wash of the demonstrator, and (**c**) cure of the demonstrator.

At the end of the printing process, as shown in Figure 15a, the part still anchored to the build plate, including the supporting structures was transferred inside the wash to remove any excess resin with isopropyl alcohol, as shown in Figure 15b.

**Figure 16:**
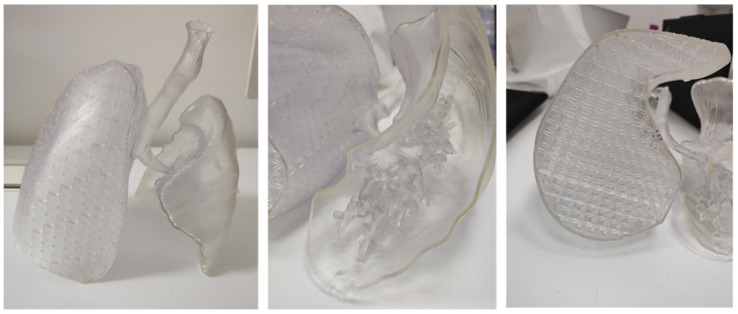
Demonstrator structure.

After the time required for washing, which for Flexible80A was 20 min, the structure was removed from the build plate and half a wash process was performed to clean the part that was prior in contact. Subsequently, the cure process was performed, in the proper oven, for 10 min, as recommended for this resin (see Figure 16). The final structure is displayed in Figure 16.

This preliminary fabrication phase was not intended as a full mechanical validation but rather as a feasibility demonstration aimed at verifying printability and assessing the tactile response of the model. Such aspects are particularly relevant in the context of surgical planning, where the ability of 3D-printed organs to provide realistic haptic feedback when integrated into surgical phantoms represents a key first step. More rigorous experimental testing will be addressed in future studies to complement these initial findings.

## 6. Sensitivity Analysis on Lattice Cell Topologies

Possible directions for improvement include the adoption of alternative lattice topologies and cell sizes. In this regard, a sensitivity analysis was carried out by varying both the geometry and the scale of the unit cells, while keeping the same resin, in order to evaluate their influence on the overall mechanical response of the model. The objective of this investigation is to identify configurations capable of better approximating the elastic behavior of real lung tissue, despite the inherent limitations of the material for 3D printing. Specifically, three different unit cell configurations were considered: the body-centered cubic (BCC), the diamond, and the re-entrant architectures. The aim of this comparison was to investigate the impact of internal microstructure on the overall mechanical response of the lung model, with the goal of approximating as closely as possible the behavior of real lung tissue. To this end, the out-of-plane displacement of a representative node located at the center of the lung geometry was monitored and used as a comparison metric.

**Figure 17:**
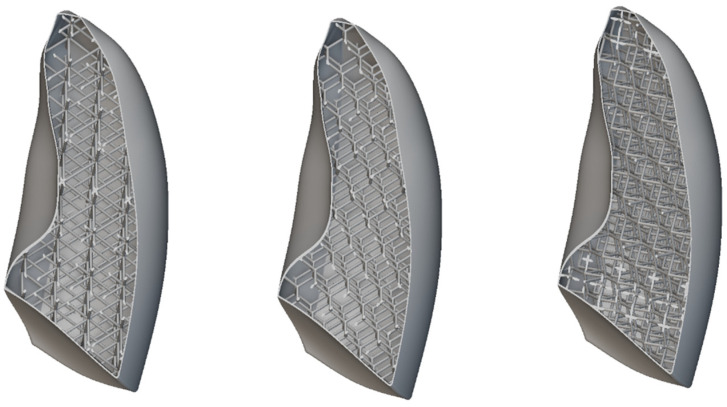
Lattice cells infill: BCC, diamond, and re-entrant.

All the considered unit cells are shown in Figure 17. In all cases, the skin thickness was 1 mm. The unit cell dimensions were 30 × 30 × 30 mm, with a consistent wall thickness of 2 mm.

**Figure 18:**
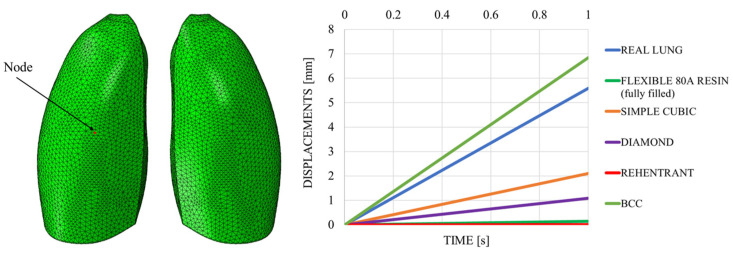
Displacements vs. time; sensitivity analysis.

**Figure 19:**
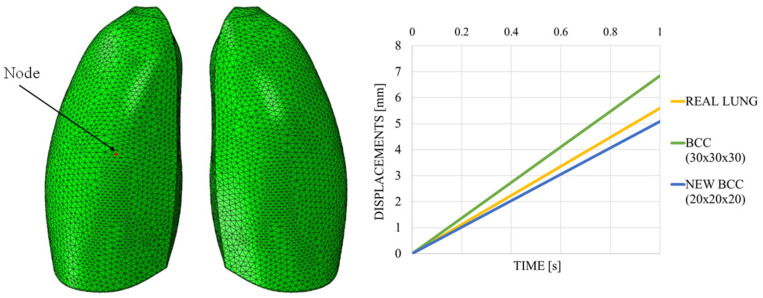
Displacements vs. time; BCC cell.

Figure 18 shows the out-of-plane displacement trend comparison measured at a representative node positioned at the center of the lung model (red dot in Figure 19), reported as a function of the typical respiratory time. The results in Figure 19 highlight notable differences in the deformation trends associated with the three cell types, offering useful insight for the selection of the most appropriate lattice architecture to better approximate the elastic behavior of real lung tissue. Among the three analyzed unit cells, the BCC cell exhibited the behavior that most closely mimics that of the lung. However, it appeared to be even more elastic, suggesting that the internal infill was too sparse and should be slightly densified. Indeed, considering cell dimensions of 20 × 20 × 20 mm, with a consistent wall thickness of 2 mm and a skin of 1 mm, the results were very close to the one obtained with the lung material model, as shown in Figure 19, which shows the out-of-plane displacement trend comparison measured at a representative node positioned at the center of the lung model (red dot in Figure 19), reported as a function of typical respiratory time.

## 7. Conclusions

This study explored the application of aerospace-derived design technologies, specifically the use of lattice structures, to faithfully replicate the mechanical and structural properties of human alveolar lung tissue. Starting with a three-dimensional CAD model of the lungs, numerical simulations were conducted using Abaqus CAE software to assess the elastic response of the lungs, both with a solid material (Flexible 80A resin) and through the integration of internal lattice structures.

The results have shown that conventional 3D printing using commercially available flexible materials is insufficient to reproduce the high elasticity of real lung tissue, which exhibits significantly greater deformability. To overcome this limitation, a lattice-based infill strategy was adopted using nTopology software, following design for additive manufacturing (DfAM) principles. The implementation of a simple cubic lattice structure resulted in a mechanical response closer to that of biological tissue, although still lower in absolute terms. Nevertheless, this approach represented a significant improvement over the solid model.

The production of prototypes through stereolithographic 3D printing further confirmed the technical feasibility of the proposed method, enabling a preliminary evaluation of the structural integrity and tactile feedback of the printed model. Finally, a sensitivity analysis involving different lattice geometries (BCC, diamond, and re-entrant) indicated that the BCC configuration, when appropriately densified, yields an elastic response that more closely approximates that of real lung tissue.

## 8. Future Directions

Future research will aim to further enhance the biomechanical fidelity of 3D-printed lung models, with particular focus on replicating the superelastic behavior and heterogeneous microarchitecture of natural lung tissue. In this preliminary study, the Flexible 80A resin was selected due to its compatibility with SLA 3D printing technology, enabling initial investigations of lattice-based lung models. Future work will explore advanced biomimetic lattice geometries, informed by high-resolution imaging and morphometric analysis of alveolar structures, to more accurately reproduce the spatial distribution of air-filled compartments and tissue stiffness. Additionally, the optimization of material systems will be pursued, including softer elastomeric resins, composite formulations, hydrogels, and multi-material printing strategies, to achieve tunable mechanical properties that better match the nonlinear elastic response of lung parenchyma. Alternative additive manufacturing technologies, such as fused filament fabrication (FFF), will also be considered to expand material compatibility and manufacturing versatility. Pre-stressing or active mechanical conditioning techniques may be implemented to simulate the dynamic cyclical expansion and contraction experienced during respiration. Computational modeling and finite element analysis will continue to guide lattice design and material selection, enabling predictive tuning of mechanical performance. Ultimately, these developments will contribute to the creation of patient-specific lung models for surgical planning, procedural simulation, and biomechanical research. Furthermore, to address the high computational cost of meshing complex lattice structures, particularly at finer resolutions or in whole-organ models, future studies will explore the use of adaptive meshing techniques and hierarchical modeling approaches, which allow maintaining high resolution in critical regions while reducing computational complexity elsewhere.

Future work will also investigate a broader range of lattice cell types, including designs that more closely resemble alveolar structures, with the aim of combining enhanced anatomical fidelity with mechanical properties that provide realistic tactile feedback in surgical training models.

## Figures and Tables

**Table 1:** Representative categories of 3D-printed medical devices.

Device Type	Representative Examples	Key Clinical Benefits	Evidence Sources
Anatomical models	Cardiac congenital models, craniofacial models	Improves 3D understanding, team planning, trainee education, and patient communication [22]	[22,25]
Surgical guides and templates	Osteotomy guides, cutting/drill guides, endodontic templates	Increased accuracy, reduced operative time, protected critical structures [28,29,30]	[28,29,30]
Patient-specific implants (PSIs)	Mandibular plates, custom orthopedic implants	Better fit, fewer intra-op modifications, potential functional/esthetic gains [23,25]	[23,25]
Prosthetics and orthoses	Custom prosthetic sockets and external prostheses	Improved comfort, individualized biomechanics, faster iteration [21]	[21]
Bioprinted scaffolds/tissues	Tissue scaffolds, pre-vascularized constructs, bioinks	Potential for regenerative therapies and personalized grafts; translational challenges remain [26]	

**Table 2:** Material properties.

Lungs’ Material Properties		Flexible 80 A Material Properties	
Young’s modulus E	7800 Pa	Young’s modulus E	3100 Pa
Poisson’s modulus ν	0.43	Poisson’s modulus ν	0.42
Density ρ	378 kg/m^3^	Density ρ	1600 kg/m^3^

## Data Availability

The raw data supporting the conclusions of this article will be made available by the authors on request.

## References

[1] Bruschi M.L., Uchida D.T., de Oliveira M.C. (2024). 3D/4D Printing of Bioadhesive Pharmaceutical Systems: Additive Manufacturing and Perspectives.

[2] Kumar N., Tyagi M., Panchal D., Singh R.P. (2024). Advances in Manufacturing and Processing of Materials: Characterization and Applications.

[3] Kumar K. (2024). Advances in Industrial Engineering in the Industry 4.0 Era.

[4] Rybicki F.J. (2021). 3D Printing in Medicine and Its Role in the COVID-19 Pandemic: Personal Protective Equipment (PPE) and Other Novel Medical and Non-Medical Devices.

[5] Bártolo P.J., Bidanda B. (2020). Bio-Materials and Prototyping Applications in Medicine.

[6] Sguinzi R., Vidal J., Poroes F., Bartolucci D.A., Litchinko A., Gossin E., Fingerhut A., Toso C., Buhler L., Egger B. (2025). 3D modeling to predict vascular involvement in resectable pancreatic adenocarcinoma. Heliyon.

[7] Hoogerbeets S.F., Roest A.A., Valverde I., Gomez-Ciriza G., Kroft L., Hazekamp M.G. (2025). Printed Models for Better Prediction of Surgery in Patients with Double Outlet Right Ventricle. Pediatr. Cardiol..

[8] Assia-Zamora S., Cortés-Cerisuelo M., Tyraskis A., Heaton N. (2025). 3D-printed model for surgical planning in congenital porto-systemic shunt: A case report. J. Pediatr. Surg. Case Rep..

[9] Grieshaber P., Schneller A., Fonseca-Escalante E., Farag M., Krey R., Czundel A., Jaschinski C., Karck M., Gorenflo M., Loukanov T. (2025). A Low-Cost Workflow to Generate Virtual and Physical Three-Dimensional Models of Cardiac Structures. World J. Pediatr. Congenit. Heart Surg..

[10] Liang S., Lee R.Z., Lim Y.G., Lim H., Misbaah F., Wan K.R. (2025). Improving Successful Cannulation of External Ventricular Drain: 3D-Printed Surgical Guide for Inexperienced Neurosurgeons. World Neurosurg..

[11] Nagpure D., Asutkar S. (2025). 3D printing in surgery: Transforming patient-specific solutions. Multidiscip. Rev..

[12] Salkind M. (1989). Aerospace materials research opportunities. Adv. Mater..

[13] Rouse D.J. (1983). Applications of Aerospace Technology in Biology and Medicine.

[14] https://bonezonepub.com/2012/12/13/aerospace-materials-and-orthopaedic-applications-opportunities-and-challenges/.

[15] Harrison D.C., Schmidt E.V., Brennan L.F. (1979). Medical Applications of Aerospace Technology. The Space Congress^®^ Proceedings.

[16] Polio S., Kundu A.N., Dougan C., Birch N., Aurian-Blajeni D., Schiffman J., Crosby A., Peyton S. (2018). Cross-platform mechanical characterization of lung tissue. PLoS ONE.

[17] Zhou B., Zhang X. (2018). Lung mass density analysis using deep neural network and lung ultrasound surface wave elastography. Ultrasonics.

[18] Mathar R.J. (2013). Hierarchical Subdivision of the Simple Cubic Lattice. arXiv.

[19] Khan N., Riccio A. (2024). A systematic review of design for additive manufacturing of aerospace lattice structures: Current trends and future directions. Prog. Aerosp. Sci..

[20] Lausted C.G., Johnson A.T., Scott W.H., Johnson M.M., Coyne K.M., Coursey D.C. (2006). Maximum static inspiratory and expiratory pressures with different lung volumes. Biomed. Eng. Online.

[21] Kermavnar T., Shannon A., O’Sullivan K.J., McCarthy C., Dunne C.P., O’Sullivan L.W. (2021). Three-dimensional printing of medical devices used directly to treat patients: A systematic review. 3D Print. Addit. Manuf..

[22] Sun Z. (2020). Clinical applications of patient-specific 3D printed models in cardiovascular disease: Current status and future directions. Biomolecules.

[23] Prządka M., Pająk W., Kleinrok J., Pec J., Michno K., Karpiński R., Baj J. (2025). Advances in 3D Printing Applications for Personalized Orthopedic Surgery: From Anatomical Modeling to Patient-Specific Implants. J. Clin. Med..

[24] Andrés-Cano P., Calvo-Haro J.A., Fillat-Gomà F., Andrés-Cano I., Perez-Mañanes R. (2021). Role of the orthopaedic surgeon in 3D printing: Current applications and legal issues for a personalized medicine. Rev. Esp. Cir. Ortop. Traumatol. (Engl. Ed.).

[25] Kattimani V., Sreeram R.R., Panga G.S.K., Vasamsetti D.B., Tiwari R. (2025). Patient-Specific Implants in Maxillo-Facial Reconstruction: Current Practices and Way Forward—An Overview of Systematic Reviews. J. Maxillofac. Oral Surg..

[26] Jovic T.H., Combellack E.J., Jessop Z.M., Whitaker I.S. (2020). 3D Bioprinting and the Future of Surgery. Front. Surg..

[27] Nasrallah A., Alqadi L., Issa A. (2024). Integrating 3D printing technology in Surgical Planning and Prosthetic Development: Current Application and Future Prospects. Arch. Med. Rep..

[28] Melhem-Elias F., Reis B.A.Q., de Oliveira N.K., Grillo R. Eminectomy performed through an innovative digital workflow. Int. J. Oral Maxillofac. Surg..

[29] Zheng C., Pan X., Peng J., Zhou X., Shi X., Yang L., Luo Y., Liu H., Zhong Z., Peng G. (2025). Primary Evaluation of Three-Dimensional Printing-Guided Endodontics in the Dog Maxillary. Vet. Sci..

[30] Paerhati W., Liu W., Wang X., Zhao B., Li F. (2025). Biomechanical characteristics and clinical application of three-dimensional printed osteotomy guide plate combined with Ilizarov technique in treatment of rigid clubfoot. Zhongguo Xiu Fu Chong Jian Wai Ke Za Zhi.

